# Observation of *η*-Al_41_Sm_5_ reveals motif-aware structural evolution in Al-Sm alloys

**DOI:** 10.1038/s41598-019-43079-9

**Published:** 2019-04-30

**Authors:** Z. Ye, F. Meng, F. Zhang, Y. Sun, L. Yang, S. H. Zhou, R. E. Napolitano, M. I. Mendelev, R. T. Ott, M. J. Kramer, C. Z. Wang, K. M. Ho

**Affiliations:** 1 Ames Laboratory, US Department of Energy, Ames, Iowa 50011 USA; 2 Department of Physics, Iowa State University, Ames, Iowa 50011 USA; 3 Hefei National Laboratory for Physical Sciences at the Microscale and Department of Physics, University of Science and Technology of China, Hefei, Anhui 230026 China; 4 Department of Materials Sci. and Eng., Iowa State University, Ames, Iowa 50011 USA

**Keywords:** Structure of solids and liquids, Physics

## Abstract

Using an effective genetic algorithm, we uncover the structure of a metastable Al_41_Sm_5_ phase that supplements its family sharing similar short-range orders. The phase evolves upon heating an amorphous Al-9.7 at.% Sm ribbon, produced by melt-spinning. The dynamical phase selection is discussed with respect to the structural connections between the short-range packing motifs in the amorphous precursor and those observed in the selected phases. The phase elucidated here is one of several newly discovered large-unit-cell phases found to form during devitrification from the glass in this binary system, further illustrating the power and efficiency of our approach, the important role of structural hierarchy in phase selection, and the richness of the metastable phase landscape accessible from the glassy structure.

## Introduction

Growing demand for advanced materials with enhanced functionality promotes expansion of the set of accessible structures. While stable materials have been efficiently identified and produced, meta-stable states are considered as a big challenge to be predicted and realized. Glass-forming alloys offer a rich landscape of non-equilibrium states, both crystalline and non-crystalline, and far-from equilibrium pathways to access them. The pathways can be manipulated through changing the starting points of materials, such as the processing parameters and the chemical composition of alloy. Al-Sm alloys, known as marginal glass formers, provide a prototypical model system where a rich collection of intermediate meta-stable crystalline phases can be accessed through path-dependent devitrification processing^1–4^. A fundamental scientific question is, what is the underlying physics mechanism of phase selections in this far-from-equilibrium system and how to control the pathways to access a myriad of meta-stable structures. A basic understanding of the physical principles that govern these pathways could very well enable application of the same principles to many different systems.

The structural orders can be a key to understanding the pathways and phase selections. Taking into account the large size and affinity disparity between Al and Sm atoms, the structural features in the Al-Sm system is most easily visualised by focusing on the the solute Sm-centered short-range order (SRO). The Al-Sm system has already exhibited a rich collection of Sm-centered ordering in known crystalline compounds, including Al_2_Sm, Al_3_Sm, Al_4_Sm and Al_11_Sm_3_^1,2,5,6^. A discussion of Sm-centered SRO in these stable compounds can be found in ref.^7^. Nevertheless, the target Al-rich system (glassy structures are attainable from 7 to 13 at.% Sm) has a different composition from the above compounds, and a new Sm-cenered ordering was found in the Al-10 at.% system^7^. This Sm-centered SRO transcends liquid, glass and crystalline states. The SRO that develops in an undercooled liquid and glass plays an important role in phase selection during devitrification processes. The amorphous Al-Sm alloys realized in melt-spun ribbon and magnetron sputtered thin films devitrify following completely different pathways. Constant-heating-rate (CHR) devitrification of Al-10.2 at% Sm ribbon exhibits a polymorphic transformation that results in a cubic *ε*-Al_60_Sm_11_ phase^8^ with a lattice parameter of ~14 Å, space group $Im\bar{3}m$ (No. 229) and with 6 unique Wyckoff positions. The thin film of the same chemical composition develops compositional inhomogeneities before the formation of fcc-Al and a hexagonal *θ*-Al_5_Sm phase (i.e. Al_20_Sm_4_ in ref.^9^) with space group *P*6_3_22. (No. 182) and with 5 Wyckoff positions. Selection is also composition dependent, and the *θ*-phase is observed as the initial crystallized phase with fcc-Al during CHR devitrification of an Al-14.1% Sm melt-spun ribbon. The *ε*-Al_60_Sm_11_ and *θ*-Al_5_Sm phases share the same Sm-centered first-shell atomic packing “3-6-6-1” motif ^7,8^ (referred to as “T6” in ref.^7^). The same 3-6-6-1 motif is found dominant in undercooled liquids, indicating a clear structural inheritance from the liquid to its devitrified crystalline phases.

We have recently developed an approach to solve for undetermined complex crystal structures observed in far-from equilibrium transitions^8,9^. The approach integrates lattice and space group information from X-ray diffraction (XRD) analysis with a genetic algorithm (GA) structural search. With this approach, we have successfully identified several previously unknown large unit-cell (LUC) structures, including the *ε*-Al_60_Sm_11_ phase^8^ and the *θ*-Al_5_Sm phase (i.e. Al_20_Sm_4_ in ref.^9^). Presently, we report on the discovery and identification of a LUC (~90 atoms/cell) tetragonal structure, termed hereafter as *η*-Al_41_Sm_5_ in this work.

The unknown phase appears as a part of a polyphase assembly of metastable phases that evolve during devitrification of an amorphous Al-9.7% Sm melt spun ribbon. The existence of common Sm-centered first-shell packing motifs in each of these LUC crystal structures (*ε*, *θ*, and *η* phases) provides clear evidence for the critical role of structural hierarchy in complex phase selection in Al-Sm alloys. The structural hierarchy is referred to as the similarity of Sm-centered short-range order between a phase and its successor phase along the devitrification pathway starting from glass. In this picture, specific short-range packing motifs which contribute to glassy behavior in undercooled liquids and amorphous solids also serve as precursors for particular crystalline phases that appear during initial stages of devitrification.

## Results

### Experimental characterization

**Figure 1:**
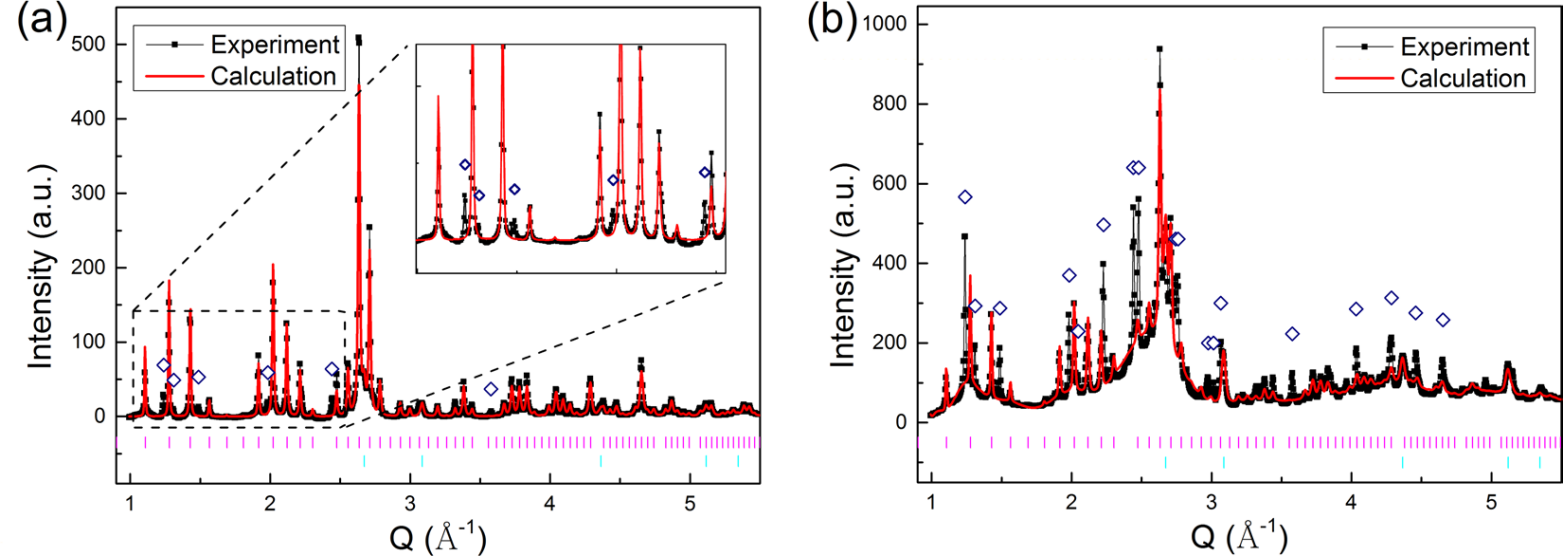
XRD patterns where (**a**) the unknown phase begins to appear, and (**b**) the unknown phase grows after an isothermal hold. Inset of (**a**): Amplified view of the low *Q* region, showing the peaks of the unknown phase. Black lines show the XRD pattern, while red lines show the Rietveld fitting of the XRD with *ε*-Al_60_Sm_11_ and fcc-Al. The vertical lines in magenta and cyan show the peak positions for *ε*-Al_60_Sm_11_ and Al, respectively. The navy diamonds indicate the diffraction peaks of the unknown phase.

**Figure 2:**
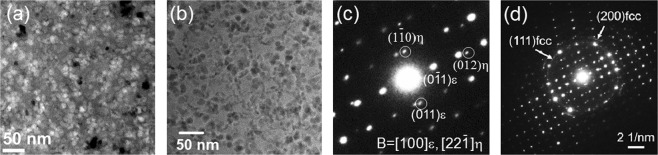
(**a**) Bright-field transmission electron microscopy and (**b**) high-angle annular dark-field (HAADF) scanning transmission electron microscopy images of a polyphased matrix of the metastable phases. (**c**) Zoom-in view of the corresponding selected area electron diffraction (SAD) pattern of the *ε*-Al_60_Sm_11_ phase and the unknown phase (designated *η*-Al_41_Sm_5_ later in this work). The zone axis is $[100]$ for the *ε*-phase, and is identified later as $[22\bar{1}]$ for the *η*-phase. (**d**) the SAD pattern that shows diffraction rings from nanocrystalline fcc-Al grains.

Upon CHR heating, the amorphous ribbon exhibits a multi-step devitrification pathway that is characterized by a series of metastable crystalline phases^2,10^. The first devitrified phases are the *ε*-Al_60_Sm_11_, fcc-Al and a small fraction of an unknown phase, as indicated by several minor XRD peaks marked by diamonds that cannot be indexed in Fig. 1(a). With an isothermal hold at 464 K, the unknown phase grows as indicated in the enhanced peaks in XRD as shown in Fig. 1(b). More peaks of the unknown phase are also observed to appear in Fig. 1(b). Figure 2(a–d) show the bright field transmission electron microscopy (BF-TEM) image, high angle annular dark field scanning transmission electron microscopy (HAADF-STEM) image and selected area diffraction (SAD) pattern, respectively. Figure 2(c) is a zoom-in view of SAD pattern in Fig. 2(d), which shows the diffraction patterns of the *ε*-phase and the unknown *η*-phase. Diffraction rings from nanocrystalline fcc-Al grains are shown in Fig. 2(d). Figure 2(c,d) suggest the grain size of the *ε*- and the *η*-phases are much larger than fcc-Al particles. However, as Fig. 2(a,b) indicate, the grain boundaries between *ε*/*ε*, *ε*/*η*, and *η*/*η* are not clearly observed, probably due to the high density of fcc-Al particles dispersed (see also Supplementary Fig. S1).

### Identification of the unknown phase

The complex structure of the unknown phase was determined by employing the approach described in ref.^8^. Using standard space group peak-matching techniques to analyze the XRD pattern shown in Fig. 1(b), we initially identify the unit cell as body-centered tetragonal with lattice parameters, a = 13.33 Å and c = 9.59 Å. Based on an assumed density equal to that of the glass (0.051 atoms/Å^3^), we estimate the number of atoms per unit cell to be approximately 90. Using a classical interatomic potential for computational expediency^11^, we perform a GA search, seeking low-energy structures with the tetragonal unit cell and the space group of *I*4 and $I\bar{4}$. The search is followed by computing an XRD pattern for each structure in the GA pool using the Rietveld program RIETAN-FP^12^ and first-principles density function theory (DFT) energy calculations^13–17^. A profile factor ${F}_{XRD}$ is calculated to assess how well the computed pattern fits the experiment measurements^8,9^. A lower ${F}_{XRD}$ indicates a better match with the experimental XRD pattern. After selecting a small set of candidate structures based on the ${F}_{XRD}$, a more accurate DFT energy is calculated^13^ for each structure.

**Figure 3:**
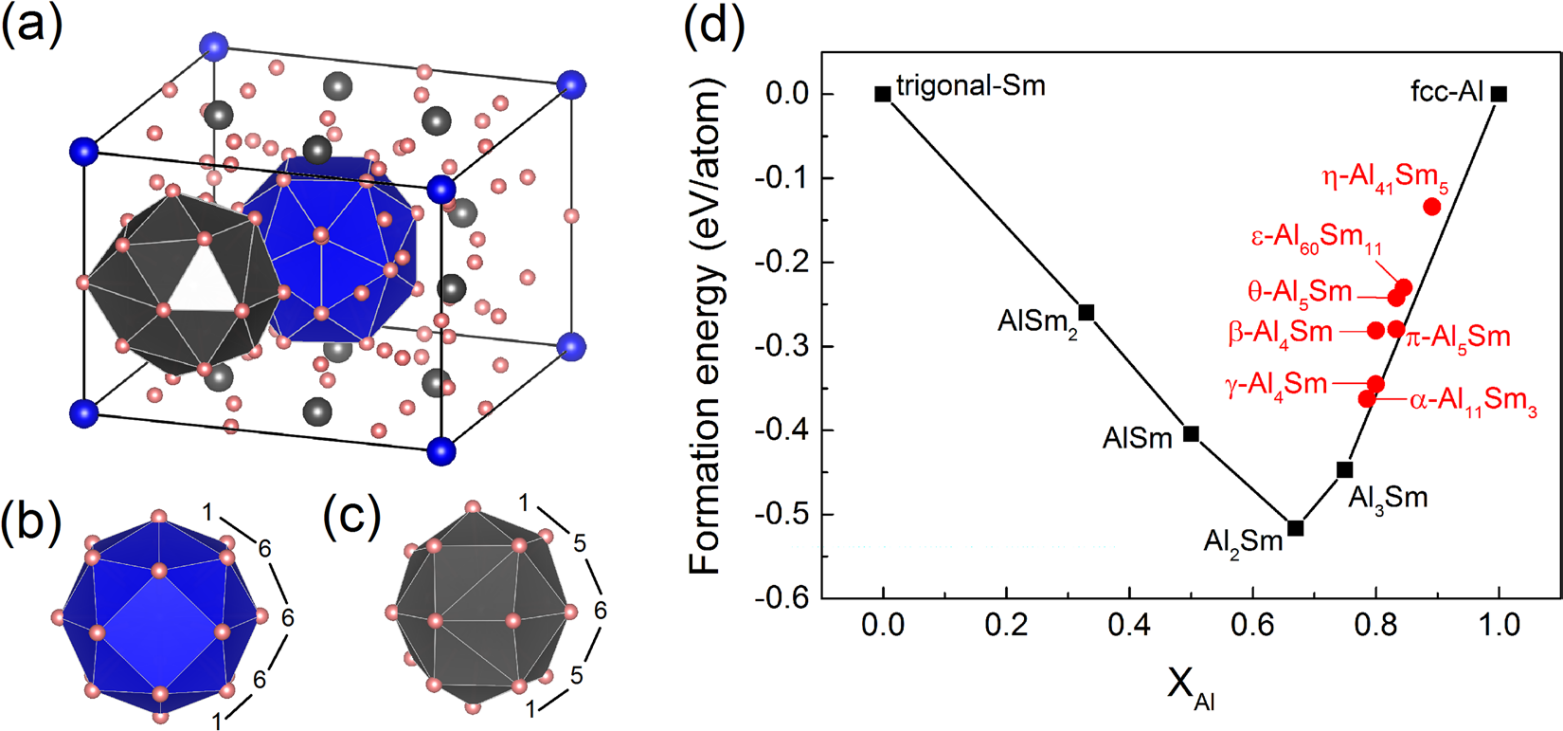
(**a**) The structure of *η*-Al_41_Sm_5_ showing two Sm-centered motifs: (**b**) the 1-6-6-6-1 motif marked in blue, and (**c**) the 1-5-6-5-1 motif in grey. Pink (blue/grey) represents Al (Sm) atoms. (**d**) The formation energy of known stable and meta-stable phases at 0 K as a function of the Al composition. The solid line connects the thermodynamically stable phases (black squares) shown in the phase diagram. The red circles are the meta-stable phases.

**Figure 4:**
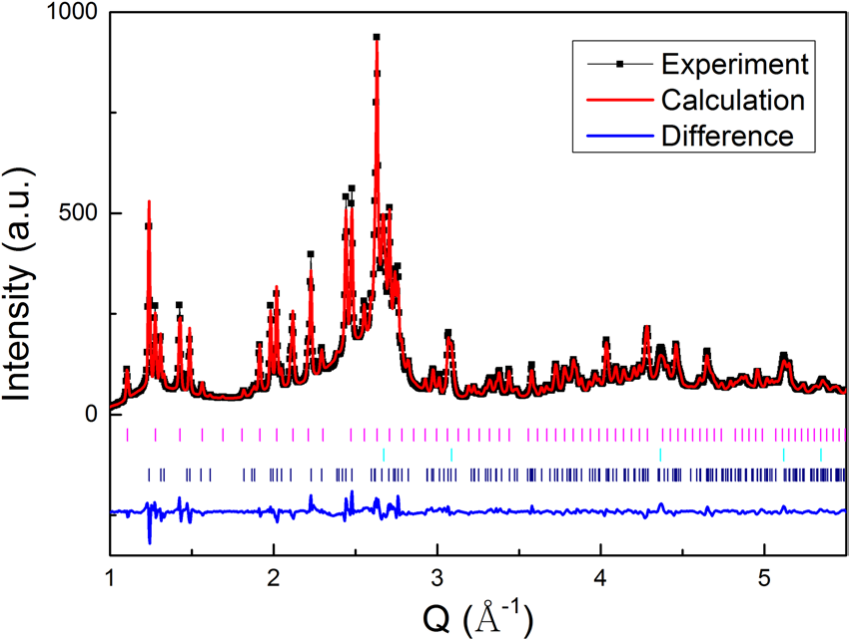
Rietveld fitting of the XRD patterns for melt spun Al-9.7% Sm ribbon at 464 K, showing the crystallization products of the cubic *ε*-Al_60_Sm_11_, fcc-Al and the tetragonal *η*-Al_41_Sm_5_. The vertical lines in magenta, cyan, and navy show the diffraction peak positions for *ε*-Al_60_Sm_11_, Al and *η*-Al_41_Sm_5_, respectively. The fitting of the experimental data gives wRp = 0.0577 and Rp = 0.0398, where wRp and Rp are the weighted and unweighted profile R-factors, respectively^26^.

There are a series of crystal structures in the GA pool with both low ${F}_{XRD}$ and low energy. Among them, the structure exhibiting the lowest formation energy is shown in Fig. 3(a). More details about other structures can be found in the discussion. The phase, designated here as *η*, exhibits a tetragonal unit cell with space group No. 87 (I4/m) including 10 Wyckoff positions and a stoichiometry of Al_82_Sm_10_. With two formula units per unit cell, the complete designation for this phase becomes *η*-Al_41_Sm_5_. The formation energy is 0.051 eV/atom with respect to fcc-Al and Al_3_Sm. It also has a low ${F}_{XRD}$, which indicates a good match with experiment XRD, as shown in Fig. 4. Figure 3(d) shows the formation energy with respect to trigonal-Sm and fcc-Al of known stable, metastable phases, and the recently solved metastable phases *π*-Al_5_Sm^4^, *ε*-Al_60_Sm_11_^8^, *θ*-Al_5_Sm^9^, and *η*-Al_41_Sm_5_. To distinguish the 2*b* and 8*h* Sm sites, they are marked in Fig. 3(a) with blue and grey, respectively. We highlight here the first shell packing environments around the 2 Sm sites, as illustrated with blue and grey polyhedral. The motif around the 2*b* and 8*h* Sm site is termed as 1-6-6-6-1 and 1-5-6-5-1, respectively, based on the packing of the atoms around the Sm atom as shown in Fig. 3(b,c).

### Rietveld refinement

A Rietveld fitting is done to refine the lattice and atomic positions. We choose to fit the data at 464 K, and the result in Fig. 4 reveals three different phases: the cubic *ε*-Al_60_Sm_11_, fcc-Al and the tetragonal *η*-Al_41_Sm_5_, which constitute ~33.3, 38.3, and 28.4 wt.%, respectively. Table 1 shows the lattice parameters and atomic coordinates of the *η*-Al_41_Sm_5_ phase, given by both DFT calculations and the Rietveld analysis.

**Table 1:** Lattice parameters and atomic coordinates of the *η*-Al_41_Sm_5_ phase with the space group No. 87 (I4/m).

Lattice parameters (in unit of Å)				
a = 13.284 (13.347), c = 9.568 (9.590)				
Atomic coordinates				
	X	Y	Z	Wyckoff
Al1	0.619 (0.610)	0.282 (0.287)	0	8 h
Al2	0.097 (0.095)	0.522 (0.516)	0	8 h
Al3	0.136 (0.138)	0.074 (0.069)	0.191(0.179)	16i
Al4	0.572 (0.568)	0.136 (0.146)	0.252 (0.244)	16i
Al5	0.564 (0.571)	0.725 (0.720)	0.145 (0.148)	16i
Al6	0.25	0.25	0.25	8 f
Al7	0	0	0	2a
Al8	0.840 (0.847)	0.282 (0.280)	0	8 h
Sm1	0.927 (0.926)	0.722 (0.723)	0	8 h
Sm2	0	0	0.5	2b

The numbers in parentheses are given by Rietveld analysis, and the rest by DFT calculations.

### Free energy calculation

**Figure 5:**
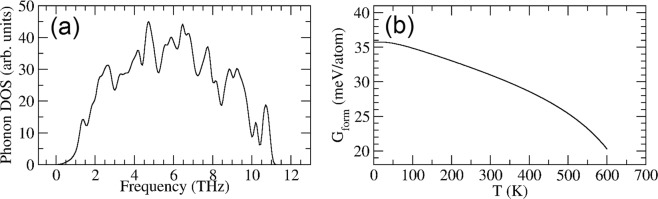
(**a**) Phonon density of states of the *η*-Al_41_Sm_5_ phase. (**b**) The formation Gibbs free energy as a function of the temperature referenced to Al and Al_3_Sm.

At 0 K, the *η*-Al_41_Sm_5_ phase is 0.051 eV/atom unstable with respect to phase separation into the Al_3_Sm phase and pure Al. To investigate the effects of finite temperatures, we calculated the Gibbs free energy energy within the quasi-harmonic approximation using the Phonopy package^18^. At a fixed volume, the Holmoltz free energy under the harmonic approximation is given by

1 $$F={E}_{0}+{k}_{B}T\sum _{nk}\,\mathrm{ln}[2\,\sinh \,\frac{\hslash {\omega }_{n}(k)}{2{k}_{B}T}],$$

where *E*_0_ is the zero-temperature total energy from VASP calculation, and ${\omega }_{n}(k)$ is the phonon spectrum. To account for thermal expansion, the phonon spectrum is calculated at various volumes, and the Gibbs free energy *G* is obtained by minimizing *F* with respect to the volume^18^. In Fig. 5(a), we first show the phonon density of states of the *η*-Al_41_Sm_5_ phase at 0 K. No negative phonon modes were observed, indicating that the *η*-Al_41_Sm_5_ phase is mechanically stable. In Fig. 5(b), we plot the formation Gibbs free energy, referenced to fcc-Al and Al_3_Sm, as a function of temperature, where one can see that the *η*-Al_41_Sm_5_ phase remains unstable w.r.t. Al and Al_3_Sm (${G}_{form} > 0$) for the entire temperature range of the devitrification process. However, ${G}_{form}$ decreases with the temperature, showing that it becomes more stable as temperature increases. It should be noted that our calculation did not fully address the aharmonicity in the system, which can have non-negligible effects at higher temperatures. Approaches that can deal with such effects, such as the thermodynamic-integration method^19^, are much more demanding computationally, and are rarely applied at the ab-initio level. Meanwhile, we believe that more accurate calculations will not change the marginally unstable nature of the *η*-phase.

## Discussion

**Figure 6:**
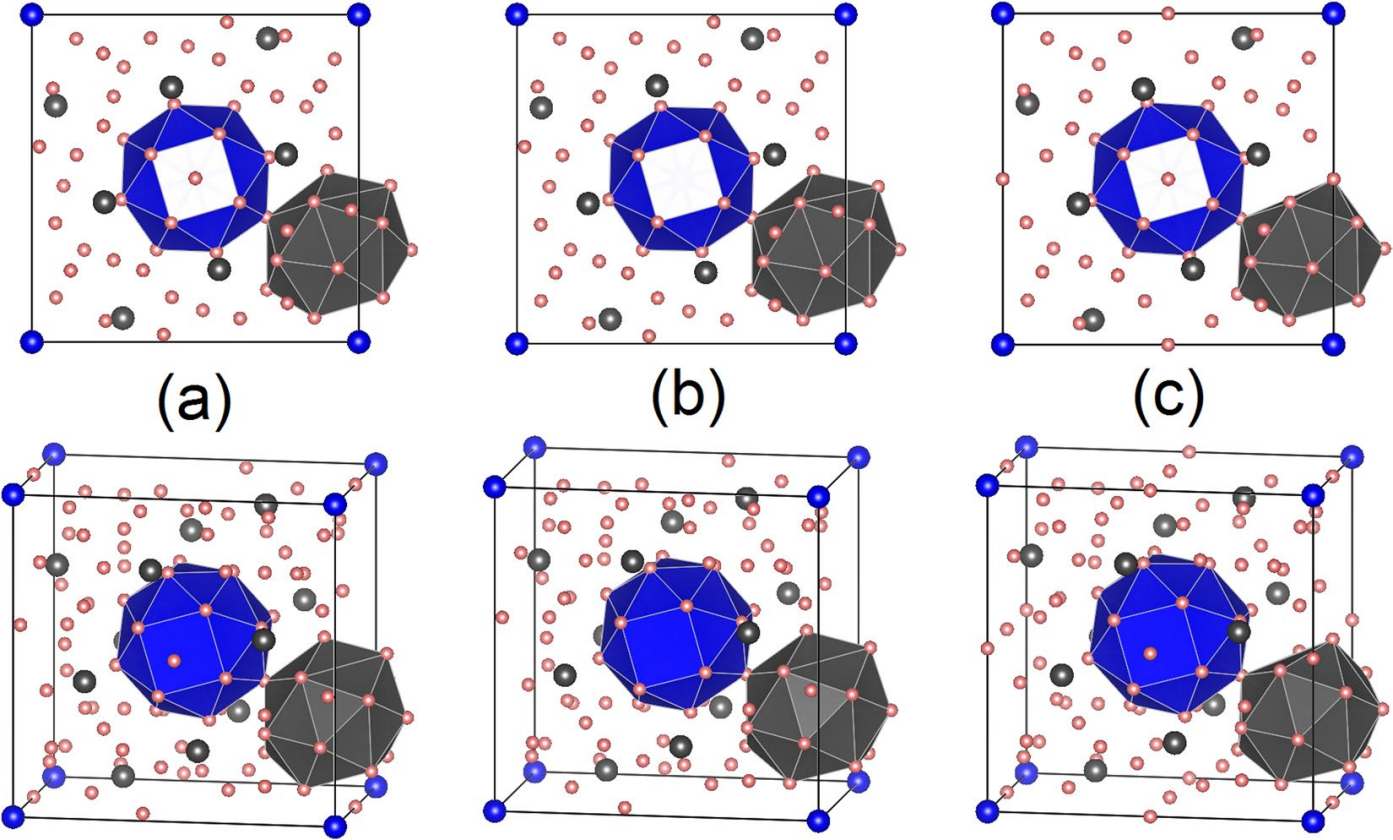
The crystal structures with lowest formation energy: (**a**) Al_82_Sm_10_ (e.g. the *η*-Al_41_Sm_5_ phase), (**b**) Al_80_Sm_10_, and (**c**) Al_78_Sm_10_ with formation energy of 0.051, 0.055, and 0.063 eV/atom respectively, w.r.t. fcc-Al and Al_3_Sm. All 3 structures have the 1-6-6-6-1 motif marked in blue. Pink (blue/grey) represents Al (Sm) atoms.

A series of similar crystal structures are found in the GA search with low ${F}_{XRD}$ and low energy. Figure 6 shows the first 3 crystal structures with the lowest formation energy. The 3 structures have a tetragonal unit cell with slightly different stoichiometries. The distribution of Sm atoms are very similar but a varience in the distribution of Al atoms is observed probabaly because the number of Al atoms are different. All the 3 structures have the 1-6-6-6-1 motif. The other motif marked in grey is somewhat different in Al_78_Sm_10_. We note that it is usually hard to determine the exact structure of a complicated crystal with a big unit cell like this. Instead a series of similar structures can be found, all of which are characterized by a common Sm-centered 1-6-6-6-1 motif. The one with the lowest formation energy, Al_82_Sm_10_ (e.g. the *η*-Al_41_Sm_5_ phase), is picked up for Rietveld refinement and free energy calculation.

**Figure 7:**
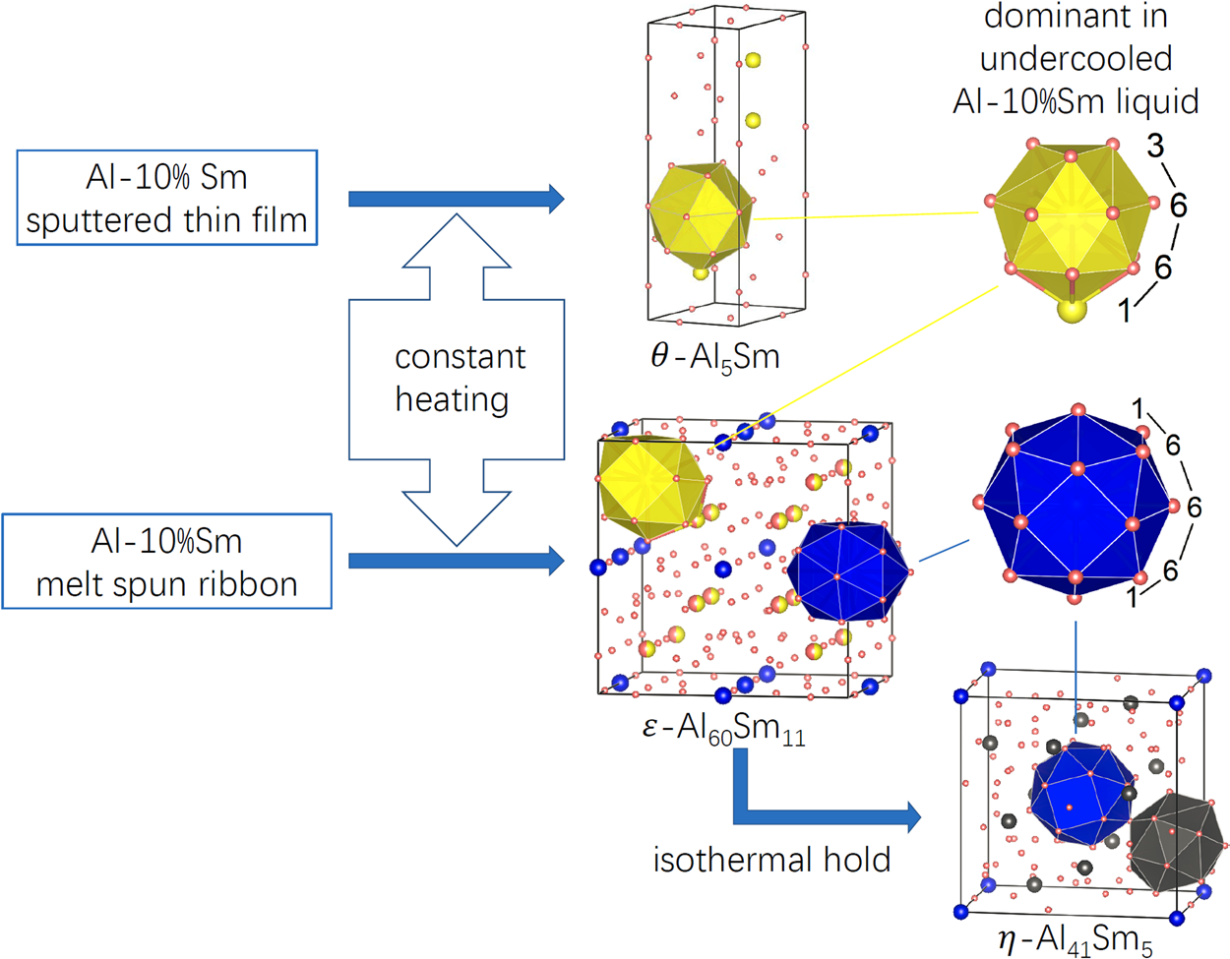
The structural hierarchy in the devitrification pathways of Al-10at.% Sm magnetron sputtered thin film and melt spun ribbon. The *θ*- and *ε*-phases share the same 3-6-6-1 motif as dominant in undercooled liquid. The *η*- and *ε*-phases share another 1-6-6-6-1 motif.

While lacking long-range translational symmetry, the as-quenched Al-Sm glasses, like many glasses, have clear elements of short range order (SRO). In particular, these Sm-centered packing motifs, play a crucial role in phase selection during devitrification. In simulated undercooled Al-10% Sm liquids, the “3-6-6-1” motif is the dominant Sm-centered motif ^7^. Experimentally the Al-Sm glasses can be synthesized by melt spinning and magnetron sputtering. As shown in Fig. 7, the *θ*-Al_5_Sm phase (along with fcc-Al) precipitates from the amorphous sputtered Al-10% Sm thin film^9^. The *θ*-Al_5_Sm structure is composed exclusively of the same “3-6-6-1” motif, indicating a well-defined structural order that transcends glass and its devitrified crystalline phase. The *ε*-Al_60_Sm_11_ phase^8^ precipitates from the amorphous melt spun Al-10.2% Sm ribbon. The *ε*-Al_60_Sm_11_ phase also exhibits the same “3-6-6-1” motif, indicating a clear structural inheritance from the glass. In addition, it has another “1-6-6-6-1” motif ^8^. The *η*-Al_41_Sm_5_ phase appears with a small fraction along with the *ε*-Al_60_Sm_11_ phase devitrified from the melt spun Al-9.7% Sm ribbon. It grows with an enhanced fraction under an isothermal hold. The *η*-Al_41_Sm_5_ phase is composed of the same “1-6-6-6-1” as in the *ε*-Al_60_Sm_11_ phase and a new “1-5-6-5-1” motif as shown in Fig. 3(a–c). The appearance of similar cluster motifs between the *ε*-Al_60_Sm_11_ and the *η*-Al_41_Sm_5_ phases provides another solid evidence for the structural hierarchy mechanism of complex phase selections in Al-Sm alloys. The question remains, however, as to whether the *η*-Al_41_Sm_5_ phase is formed directly from the glass or the *ε*-Al_60_Sm_11_ phase is a precursor for the formation of the *η*-Al_41_Sm_5_ phase. It is open to question as well why the sputtered or the melt-spun material would prefer the formation of a particular phase instead of others. The medium-range order (MRO), or how the local motifs are packed at medium range, may also strongly affect the complex phase selection. Different MROs are observed with different processing methods or thermal treatments. A laser pretreatment changes MRO in Ag/In-incorporated Sb_2_Te^20^. Magnetron-sputtered and pure ion-implanted amorphous silicon exhibit significantly different SRO and MRO following thermal annealing^21^. Different distribution of icosahedral- and crystal-like superclusters are observed with varying annealing temperature in simulated Zr_50_Cu_45_Al_5_ bulk metallic glass^22^. While these studies have advanced our comprehension of structural order in amorphous materials, detailed discussion of MRO is beyond the scope of this work.

In summary, we observed an unknown metastable phase during the devitrification of melt spun Al-10% Sm glasses. Using an efficient genetic algorithm combined with experimental diffraction data, we found a series of similar crystal structures which have low energy and fit well with the experiment XRD. The crystalline phase with the lowest formation energy, termed *η*-Al_41_Sm_5_, has a big tetragonal unit cell that contains 92 atoms with the stoichiometry Al_82_Sm_10_. The calculated X-ray diffraction pattern matches well with that of the experiments. *η*-Al_41_Sm_5_ is mechanically stable from phonon spectrum calculation. It is metastable with respect to phase separation into Al_3_Sm and pure Al at finite temperatures. Resolution of the atomic structure of these new metastable complex crystal phases lay the groundwork for further investigations to elucidate how different processing protocols can influence the selection and growth of different metastable crystal phases in the devitrification process. Examining the 3 metastable phases observed so far in devitrification experiments, we find a common picture emerging where complex metastable phases which appear have structures dominated by specific atomic clusters (motifs) centered about Sm atoms. This supports structure hierarchy picture of complex phase formations and suggests a possible physical mechanism where the low mobility of Sm atoms during devitrification process plays an important role in the selection of metastable crystal phases compatible with certain Sm-centered cluster motifs.

## Methods

### Preparation of the amorphous alloy

Alloys of Al-9.7% Sm were prepared in ingots of 10 grams by simultaneously arc-melting the pure components (99.99 wt% Al and 99.9 wt% Sm) under an Ar atmosphere. The alloy ingots were re-melted under 1/3 atm argon and rapidly solidified into ribbons (0.02–0.03 mm thick) by free-jet melt-spinning onto a rotating Cu wheel (tangential speed of 30 m/s). Both high resolution transmission electron microscopy (TEM) and high energy X-ray diffraction (HEXRD) reveal the amorphous nature without any detectable crystallized phase (not shown here).

### Crystallization of the amorphous alloy

Crystallization of the amorphous alloy ribbons was investigated using time-resolved synchrotron-based HEXRD (71.77 keV energy, 0.1729 Å wavelength), utilizing the 1-ID-E beamline of the Advanced Photon Source (APS) at Argonne National Laboratory. Specimens for HEXRD were prepared by cutting melt-spun ribbon into lengths of approximately 10 mm, stacking multiple segments to a thickness of ~0.5 mm, and inserting into a 2 mm ID thin walled SiO_2_ capillary tube which was sealed under argon. An infrared heater was used for *in-situ* heating and isothermal holding. HEXRD pattern revealed that the *ε*-Al_60_Sm_11_ phase and a small amount of fcc-Al were the first phases to appear during devitrification. There were several minor XRD peaks that cannot be indexed, as marked by diamonds in Fig. 1(a), suggesting a small fraction of an unknown phase mixed with the *ε*-Al_60_Sm_11_ phase and fcc-Al. With an isothermal hold at 464 K (about 5 K lower than the onset temperature for crystallization) for 70 mins, the unknown phase grew as indicated in the enhanced peaks in XRD as shown in Fig. 1(b). Post-devitrification samples were analyzed using TEM (Tecnai G^2^ F20). TEM specimens were prepared using a dual-beam focused ion beam (FIB) instrument (FEI Helios NanoLab G3 UC). BF-TEM, HAADF-STEM images and SAD pattern (Fig. 2(a–d)) were obtained to analyze the post-devitrification samples. The BF-TEM image shown in Fig. 2(a) and the HAADF-STEM image in Fig. 2(b) clearly demonstrated a fully crystallized polyphase material. The nanocrystals uniformly distributed in the matrix with grain size of ~20–30 *nm* in Fig. 2(a) were fcc*-*Al, which corresponded to the dark region (low *z*-contrast) in HAADF-STEM image of Fig. 2(b).

### Genetic algorithm (GA)

A GA was used to search for low energy structures by defining the fitness as a function of energy. All structure relaxations during the GA search were performed by LAMMPS code^23^ with Embedded-Atom Method (EAM) potential in Finnis-Sinclair form^24^. As previously shown^4,11^, this FS potential fitted to first principles calculation data, in general, gives a satisfactory estimation of the relative thermodynamic stability of the known stable and meta-stable phases.

### Density Functional Theory (DFT)

After a GA search for low energy structures using EAM potential, a more accurate DFT energy was calculated for a selection of structures which are a good match with experiment XRD. All DFT calculations were performed using the Vienna *ab initio* simulation package (VASP)^14^ with the projector-augmented wave (PAW) pseudopotential method^15,16^ and the generalized-gradient approximation (GGA)^17^.

### Fitting XRD pattern

A standard Rietveld analysis was carried out using the GSAS package and the EXPGUI interface^25^ to refine the *η*-Al_41_Sm_5_ structure from GA search and DFT calculation. Lattice parameter, atomic coordinates, site occupancies, thermal parameters, and peak shape profiles are refined to get the XRD pattern in Fig. 4. Lattice parameters and atomic coordinates given by DFT calculations and the Rietveld analysis are provided in Table 1.

## Supplementary information

Supplementary Information for Observation of n2;-Al41Sm5 reveals motif-aware structural evolution in Al-Sm alloys
