# Supplementary material for: Observation of η-Al41Sm5 reveals motif-aware structural evolution in Al-Sm alloys
Source: Sci Rep. 2019 Apr 30;9:6692. doi: 10.1038/s41598-019-43079-9 (PMC6491476; doi:10.1038/s41598-019-43079-9)
Supplement: Supplementary file 1 — Supplementary Information for Observation of n2;-Al41Sm5 reveals motif-aware structural evolution in Al-Sm alloys [file 41598_2019_43079_MOESM1_ESM.pdf]

Supplementary Information for "Observation of η-$Al_{41}Sm_5$ reveals motif-aware structural evolution in Al-Sm alloys"

Z. Ye[1*], F. Meng[1], F. Zhang[1], Y. Sun[1,3], L. Yang[1], S. H. Zhou[1], R. E. Napolitano[1,4], M. I. Mendelev[1], R. T. Ott[1], M. J. Kramer[1], C. Z. Wang[1], K. M. Ho[1,2,3*]

[1]Ames Laboratory, US Department of Energy, Ames, Iowa 50011, USA

[2]Department of Physics, Iowa State University, Ames, Iowa 50011, USA

[3]Hefei National Laboratory for Physical Sciences at the Microscale and Department of Physics, University of Science and Technology of China, Hefei, Anhui 230026, China

[4]Department of Materials Sci. and Eng., Iowa State University, Ames, Iowa, 50011, USA

Authors to whom correspondence should be addressed:

[*] Email address: zye@iastate.edu (Z.Y.); kmh@iastate.edu (K.M.H.)

Figure 2(c) and (d) suggest the grain size of the ε-$Al_{60}Sm_{11}$ and the η-$Al_{41}Sm_5$ phases are much larger than fcc-Al particles. However, as Fig. 2(a)(b) and Fig. S1(a)(b) indicate, the grain boundaries between ε/ε, ε/η, and η/η are not clearly observed, probably due to the high density of fcc-Al particles dispersed.

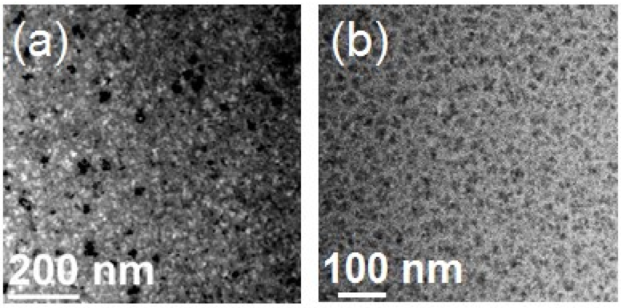

Figure S1. (a) Bright-field and (b) High-angle annular dark-field scanning transmission electron microscopy images shows the microstructure with multi-phases in Al-10.2at%Sm alloy annealed at 473K for 1800s.
